# Acquired Factor VIII Inhibitor in a Patient of Rheumatoid Arthritis on Tumor Necrosis Factor Inhibitor Therapy

**DOI:** 10.1007/s12288-018-1030-1

**Published:** 2018-10-16

**Authors:** Tomoyo Mori, Naoki Watanabe, Hideaki Kitahara, Noriaki Iwao, Michiaki Koike, Norio Komatsu

**Affiliations:** 1 Division of Hematology, Department of Medicine, Juntendo University School of Medicine Shizuoka Hospital, 1129 Nagaoka, Izunokuni City, Shizuoka 410-2295 Japan; 2 Division of Hematology, Department of Medicine, Juntendo University School of Medicine, Tokyo, Japan

Dear Editor,

Acquired hemophilia A (AHA) is a rare bleeding disorder caused by an autoantibody to factor VIII (FVIII), with an estimated incidence of 1.48/million/year. The mortality rate has been reported to be between 8 and 22% [1]. Major associations with AHA are postpartum hemorrhage, malignancy, autoimmune disorder, rheumatoid arthritis (RA), and the use of drugs (penicillin, sulfamide, and interferon alpha). In our patient, AHA occurred during tumor necrosis factor alpha (TNF-α) inhibitor treatment for rheumatoid arthritis.

A 68-year-old female was diagnosed with RA 10 years earlier, and has been treated with etanercept (tumor necrosis factor alpha (TNF-α) inhibitor) for over 6 years, at 25 mg once every week. She became aware of bruising on her left hip 3 months ago, and whole-body computed tomography (CT) revealed a large muscular hemorrhage. The serum hemoglobin level had decreased to 6.6 g/dL. The area of muscular hemorrhage of her left hip subsequently enlarged, and she was transported to our hospital in hemorrhagic shock. The results of coagulation tests showed a prolonged activated partial prothrombin time (APTT) (86.8 s, normal range 25–40) with a normal prothrombin time (PT) (13.3 s, normal range 11.3–13.3). In addition, a coagulation factor assay revealed decreased levels of FVIII activity of less than 1% (normal range 78.0–165.0%) and a high titer of FVIII inhibitor of 213 Bethesda units/mL (BU/mL). Therefore, she was diagnosed with acquired hemophilia A (AHA). Whole body CT was performed after hospitalization, and no tumors were detected.

She was administered activated prothrombin complex concentrate for the treatment of bleeding. However, new hemorrhage sites developed in her left shoulder and right leg. She was then administered recombinant activated factor VII for the treatment of bleeding. Immunosuppressive therapy was started with oral prednisolone (PSL) at 45 mg/day. However, APTT and the FVIII inhibitor level rose to 150 s and 569 BU/mL, respectively. She was administered cyclophosphamide (CPA) at 50 mg/day and then received CPA pulse therapy (500 mg/body every 4 weeks). The bleeding in soft tissue repeated, and she was administered cyclosporine A at 200 mg/day. The results of coagulation studies showed a normal APTT and disappearance of the FVIII inhibitor 15 weeks after the immunosuppressive therapy. PSL was tapered to 10 mg/day and she was discharged from our hospital on day 150. After discharge, the patient was administrated 10 mg of prednisolone every day continuously. However, after 1 year, the inhibitor was re-redacted. The administration of cyclosporine A was started again.

In our patient, AHA occurred during TNF-α inhibitor treatment, and this treatment has been reported to be associated with a high-titer of FVIII inhibitor [2]. In our case, a coagulation factor assay revealed a high-titer of FVIII inhibitor, at 569 BU/mL. It has been reported that it is difficult for AHA patients with a high titer of FVIII inhibitor to achieve complete sustained remission and that they require long-term treatment. Autoantibody to FVIII mainly consists of IgG. In addition, high-titer inhibitors and inhibitors of FVIII in patients with AHA and underlying disease consist predominantly of IgG4 [3]. A study using TNF-α knockout mice reported that the mice showed increased levels of interleukin (IL)-5 and IL-10 [4]. These cytokines have been reported to promote IgG4 production [5]. Therefore, it is suggested that TNF-α inhibitor increased IL-5, IL-10, and IgG4 production and induced the high titer of FVIII inhibitor. This is the first report that TNF-α inhibitor use may be associated with high-titer FVIII inhibitor AHA. A limitation of this study was that we did not measure IL, IgG, or IgG4. However, it was reported that IgG4 was high in high-titer AHA. FVIII inhibitor is classified as the type 1 or type 2 based on the inhibitor pattern. The inhibitor of AHA is considered a type 2 inhibitor. Additionally, the inhibitor assay can be technically challenging, especially in the context of high-titer inhibitors where errors in preparing dilutions can markedly affect the numerical value ascribed to the Bethesda units. Also, the relationship between the inhibitor concentration and effect on residual FVIII activity is not perfectly log-linear for type 2 inhibitors seen in AHA [6]. The total Naranjo Scale was 3 (possible adverse drug reaction) in this patient. However there is some room for doubt as to the adverse drug reaction to etanercept. Although further studies are needed, we consider that TNF-α inhibitor use may be associated with a high titer.

In conclusion, we report a case of high-titer FVIII inhibitor AHA successfully treated with multi-immunosuppressive drugs. We consider that TNF-α inhibitor may have been associated with high-titer AHA in the present patient.
